# Resisting and disrupting HIV-related stigma: a photovoice study

**DOI:** 10.1186/s12889-023-16741-1

**Published:** 2023-10-21

**Authors:** Gayle Restall, Patricia Ukoli, Punam Mehta, Elizabeth Hydesmith, Mike Payne

**Affiliations:** 1 Department of Occupational Therapy, Faculty of Health Sciences, University of Manitoba, Winnipeg, MB R3E 0T6 Canada; 2 Faculty of Social Work, University of Manitoba, Winnipeg, MB Canada; 3 Department of Community Health Sciences, Faculty of Health Sciences, University of Manitoba, Winnipeg, MB Canada; 4 Department of Anthropology, Faculty of Arts, University of Manitoba, Winnipeg, MB Canada; 5 Nine Circles Community Health Centre, Winnipeg, MB Canada

**Keywords:** HIV stigma, Oppression, Resistance, Qualitative research

## Abstract

**Background:**

The stigma associated with human immunodeficiency virus (HIV) is a significant global public health concern. Health care providers and policy makers continue to struggle with understanding and implementing strategies to reduce HIV-related stigma in particular contexts and at the intersections of additional oppressions. Perspectives and direction from people living with HIV are imperative.

**Methods:**

In this project we amplified the voices of people living with HIV about their experiences of HIV-related stigma in Manitoba, Canada. We used an arts-based qualitative case study research design using photovoice and narrative interviews. Adults living with HIV participated by taking pictures that represented their stigma experiences. The photos were a catalyst for conversations about HIV and stigma during follow-up individual narrative interviews. Journaling provided opportunities for participants to reflect on their experiences of, and resistance to, stigma. Interviews were audio recorded and transcribed. Photos, journals, and transcribed interviews were analyzed using inductive qualitative methods

**Results:**

Through pictures and dialogue, participants (*N* = 11; 64% women) expressed the emotional and social impacts of stigmas that were created and supported by oppressive structures and interpersonal attitudes and behaviours. These experiences were compounded by intersecting forms of oppression including racism, sexism, and homophobia. Participants also relayed stories of their personal strategies and transitions toward confronting stigma. Strategies were themed as caring for oneself, caring for children and pets, reconstituting social support networks, and resisting and disrupting stigma. Participants made important recommendations for system and policy change.

**Conclusions:**

These stories of oppression and resistance can inspire action to reduce HIV-related stigma. People living with HIV can consider the strategies to confront stigma that were shared in these stories. Health care providers and policy makers can take concerted actions to support peoples’ transitions to resisting stigmas. They can facilitate supportive and anti-oppressive health and social service systems that address medical care as well as basic needs for food, shelter, income, and positive social and community connections.

## Background

The stigma associated with human immunodeficiency virus (HIV) is a significant global public health concern [1]. HIV-related stigma has persisted despite acknowledgement of its negative impacts on people’s lives and society. Systematic reviews have highlighted attempts to reduce stigma and its impacts, primarily through education, skill building, and support [2–4]. Although some stigma reduction strategies have demonstrated success, the evidence is not robust and lived experiences confirm the persistence of stigma. The ability of HIV-related stigma to continue to perpetuate prejudice and discrimination toward people living with HIV is exacerbated by the intersectionality of additional forms of stigma and discrimination created and maintained through colonial, social, and political structures. Racism, sexism, and homophobia [4], along with additional social structures and attitudes that place people at increased risk for HIV, contribute to the intractability of the stigma experienced by people living with HIV. Greater investments in stigma reduction is required to achieve the World Health Organization 2030 targets to end the AIDS epidemic [5].

Research has been accumulating on the experiences of HIV-related stigma and its negative impacts on health and well-being [6–8]. Photovoice has been recognized as an important way for adults living with HIV to share their experiences of stigma [9]. Greater opportunities for adults living with HIV to share their stigma experiences while highlighting what is required to resist and disrupt stigma can provide guidance for system and structural change. In this community-based case study research we amplified the voices of people living with HIV through photovoice methodology to learn how participants experienced and built resistance to stigma. We identified the actions that health and social services need to take to disrupt stigma and improve the health and well-being of people living with HIV.

### The impact of stigma

HIV stigma has been described in various ways. The mechanisms by which stigma affects individuals have been conceptualized as functioning through enacted, anticipated, and internalized stigma [10]. Enacted stigma includes discriminatory behaviour toward people with a stigmatized health condition and encompasses all the ways people have experienced discrimination through violence, exclusion, blame, and additional behaviors that place them at a disadvantage socially, economically, and in their pursuit of health and well-being [11–13]. Anticipated stigma refers to the extent to which people expect they will experience prejudice and discrimination from other people [14, 15]. Internalized stigma includes negative attitudes and feelings that someone with HIV may have that are directed toward themselves [16, 17].

The extent to which people living with HIV experience stigma and how they react to stigma varies considerably. However, a body of research has demonstrated the profound negative effects of all three dimensions of stigma. For example, enacted stigma can increase the likelihood that people will experience violence [18–20], social rejection [20], and exclusion from rights and opportunities that contribute to lower rates of employment [21], housing insecurity [22] and unmet health and social care needs [23, 24]. Anticipated and internalized stigma can reduce treatment adherence [25] and increase people’s reluctance to disclose their HIV positive status creating barriers to obtaining social supports, including health care [26]. Anticipated stigma has been negatively associated the willingness to seek life opportunities such as employment [27]. Internalized stigma has been associated with a lower likelihood of initiating anti-retroviral therapy [28], more mental health concerns [29], and poorer overall self-reported health [6]. The interactions of these dimensions of stigma are complex. Turan and colleagues [26] illustrated the ways that perceived community stigma (i.e. the degree to which people with HIV perceive HIV-related stigma in their communities) can influence internalized and anticipated stigma and their effects on medical and social outcomes. Thus, the dimensions of stigma influence each other in their scope, expression, and outcomes across a variety of contexts and life situations [30].

In the context of HIV-related stigma and its variable effects on people’s lives we wanted to better understand how people with HIV live with stigma. The purpose of this study was to explore: a) the ways that people with HIV experienced stigma and its emotional and social effects, b) how they strive toward well-being to resist and disrupt stigma, and c) the social and structural supports and resources that can promote living well with HIV.

## Methods

### Design

This study used an arts-based qualitative case study [31] research design using photovoice [32] and narrative interviews [33]. Photovoice methodology facilitates communication about everyday experiences and promotes critical discussion about those experiences to influence public policy [32]. Photovoice has been used as a community-based research approach for diverse areas of concerns, priorities, and promotion of practice and policy change affecting people living with HIV [9], including examining the experiences of HIV-related stigma [34, 35]. Through photovoice, participants take photos that become a catalyst for the narration of their stories during subsequent interviews. In this way, they take more control of the interview than traditional interview methods because they determine the pictures they take and the stories they tell related to the pictures. The methodology also promotes inclusiveness through communication of experience in pictures as well as words.

### Case

The research was bounded by a particular case, that of adults living with HIV in Manitoba, Canada. Although exact numbers of people living with HIV in the jurisdiction are difficult to determine, McClarty and colleagues reported that in the first quarter of 2019 there were 1357 clients of the Manitoba HIV Program, the primary provider of HIV treatment, care and support in the province [36]. The largest proportion (64.2%) of clients were aged 40 to 64 years. Distribution of self-identified ethnicity was reported as 36.2% White, 39.9% Indigenous, and 19.1% Sub-Sahara African/Caribbean/Black. The client population was predominantly male at 64.7%. However, there have been greater proportions of females being diagnosed in recent years [37]. Self-reported potential risk exposure for both females and males newly diagnosed in the jurisdiction in 2020 was highest in people who inject drugs followed by heterosexual sex [37]. The impact of the social determinants of health has been evident in, for example, the relationship between HIV and lower income in both urban and rural areas, with a high prevalence of people who live with HIV being in the lowest income quintile [38]. Despite Manitoba having the second highest rate of HIV infections among the Canadian provinces and territories [39], there has been little research that has explored the ways that people living in the province have built resistance to their experiences of stigma.

### Participants

We purposefully recruited people, 18 years of age and older, who had been diagnosed with HIV a minimum of one year previously and were living in Manitoba. Participants had to be able to communicate in English. The research was approved by a local university Research Ethics Board. All participants provided informed consent, either in writing or recorded and documented via audio consent necessitated by social distancing public health orders due to the COVID-19 pandemic.

### Recruitment

We recruited participants through: posters at a community health centre specializing in the care, treatment, and support of people living with HIV; by contacting people who had participated in a previous study on HIV-related stigma and had agreed to be contacted for future research; through existing networks; and word-of-mouth.

### Procedures

We began the study in February 2020 with a small group of six potential participants who attended a group orientation session lasting approximately two hours in which members of the research team explained the purpose of the research, confidentially collected demographic and background information related to age, citizenship status, ethnic and racial identity, sex at birth, gender, sexual orientation, and years since HIV diagnosis. Participants were provided with training on photovoice, picture taking, use of a camera, ethics of picture taking, and safety considerations. Participants were asked to take a minimum of five pictures and to journal the meaning of the photograph using a version of the SHOWED technique described by Shaffer and reported by Catalani and Minkler [40]. Journaling prompts were: what is seen; what is happening; how does this relate to our/my life; why are things this way; how could this image educate people; and what can I do about it. We asked participants to take pictures of their experiences of stigma and provided the following suggested topics: what stigma is; why stigma exists; how to personally overcome stigma; and how to reduce systemic stigma. We gave participants great latitude on what topics they felt were most important to represent in their pictures and discuss in their follow-up interview. Although we had a general discussion about stigma in the photovoice training session, we did not tie participants to a particular definition of stigma.

Public health orders related to the COVID-19 pandemic that were implemented shortly after the first group session prevented further in-person research processes. One participant from the initial group did not continue after the orientation session. Orientation sessions for an additional six participants and all subsequent follow-up interviews were done individually via virtual teleconferencing software. In one case the individual’s partner was in the background during sessions, at the participant’s request.

Follow-up interviews were conducted once a participant had gathered at least five pictures. Interviews were facilitated by the research coordinator with support from either a peer research assistant or principal investigator. Semi-structured interviews were guided by a modified version of the SHOWED framework, as described above, with prompts to relate the conversation specifically to stigma. Participants were also asked about key messages for people living with HIV, service providers, and policymakers. The interviews were audio-recorded and transcribed verbatim.

### Analysis

Photos, journals, and interview transcripts were sources of data that were analysed using inductive thematic qualitative methods adapted from Miles, Huberman, and Saldana [41] and following the traditions of Braun and Clarke [42]. Inductive thematic analysis has been employed in previous photovoice studies [43]. It was chosen as the analytical approach because of its flexibility. It also places emphasis on participants’ interpretations, in this case as elicited through their photos, journals, and individual interviews, rather than relying on the researcher’s theoretical perspective [42]. In addition, it is consistent with the researchers’ social justice approach demonstrated by forefronting the perspectives of participants as they spoke about the ways that stigma impacted their lives and their perspectives of remedies for social stigmatization.

Two team members were involved in data analysis. One team member began first cycle analysis by developing a basic coding structure through line-by-line coding of the first interview transcript in the context of the photos and journal. A second team member, who became the primary data analyst, read the transcript and reviewed the coding structure. The two team members met to discuss the coding and interpretations. Next, the primary analyst conducted first cycle analysis on all subsequent transcripts through line-by-line coding using the initial coding structure and adding new codes as they were conceptualized from the data. The two team members met on a regular basis during the coding process to discuss codes and interpretations. The primary analyst noted salient points through memo writing [40]. Definitions of codes were refined and discrepancies in interpretations resolved through discussion. Next, second cycle pattern analysis involved the primary analyst searching for and reviewing themes [42] by grouping first cycle codes into larger thematic categories. These larger categories were refined and reorganized by the two team members with attention to identifying cross case confirming and disconfirming comparisons [41]. Themes and subthemes were defined and named [42]. Photos and journals were consulted iteratively during the process of describing the themes.

## Results

Eleven [11] participants completed the photovoice process. Table 1 summarizes their demographic and social background information. All participants identified as cis-gender.

**Table 1:** Participant demographics

Variable	Frequency
Age (years)	
*M* = 46 (*SD* = 10.35) (Range: 36—64)	
Length of time since diagnosis (years)	
*M* = 14 (*SD* = 6.71) (Range: 6—27)	
Sex/Gender	
Male/Man	4
Female/Woman	7
Sexual orientation	
Heterosexual	6
Bisexual	2
Gay	2
Prefer not to answer	1
Ethnic or racial group (self-identity)	
Indigenous	4
White	3
Black or African	3
Asian/White	1

In this manuscript we highlight the ways that participants strove for a good life in which they could experience well-being in the face of HIV-related stigma. All participants shared the emotional pain and social rejection precipitated by HIV-related stigma. Yet, they found new ways of living a life. Although stigma continued to create barriers to their well-being, they shared stories of their inner strengths and resources, as well as external factors that helped them along that journey.

We begin by describing the *experiences of intersecting stigmas and the emotional and social impacts of stigma* that participants chose to share to provide context for the factors that supported participants as they were *striving toward well-being*. We then describe the four themes that emerged about the factors that supported well-being a) *caring for oneself,* b) *caring for children and pets*; c) *reconstituting social support networks*; and d) *resisting and disrupting stigma*. We end by summarizing the system and structural resources that participants identified as important to flourishing in the context of HIV stigma. Quotes are identified according to the participant’s gender woman (W) or man (M) and a code number.

### Experiences of intersecting stigmas

Although participants were asked about their experiences of HIV-related stigma, for many, these experiences could not be disengaged from the intersectionality of stigma associated with additional social identities. Stories of intersecting stigmas focused on racism, sexism, and homophobia.

Racism was identified by several participants as having significant negative effects on their lives both prior to, and after, being diagnosed with HIV. For example, one participant spoke about the verbal abuse and social marginalization experienced as a Black person growing up in a predominately White community and how that prompted them to leave school and the community. Indigenous participants talked about being bullied and verbally abused. Another participant explained how being Black, and an immigrant to Canada both intersected with HIV in their experience of stigma and discrimination.

Sexism was described in the context of stigma and discrimination by two women participants who noted the challenges that women often faced. They identified attitudes and behaviours focused on blaming women. One participant noted that:

There is an idea that women ask for it, you know, that they ask to be in… abusive relationships…. There is a perverse idea that they have to explain themselves. You know, otherwise, they just must fit into a category. It must be their fault” (W03).

Another participant who was an immigrant to Canada shared her perspective of how women in some cultures can be blamed in particular ways for anything that is perceived as wrong, leading to abuse and being trapped in marriages with no way out. Another woman spoke about prevailing assumptions that people who are diagnosed with HIV must be either street workers or injection drug users. These assumptions were connected to a blaming culture inside and outside health and social care systems that tied HIV infection to people’s personal behaviours rather than the structural systems that placed people at risk.

Homophobia was also a source of discrimination. Participants who identified as gay talked about the ways that having HIV compounded negative attitudes from family and other social networks. One participant said:

Well you know, for me it [is my] family that doesn’t respect me, … like my mother is embarrassed about me. It was bad enough that I was gay, but HIV was a complete no, no. She loved me anyways, but she was embarrassed, and it was shameful [for her] (M09).

For this participant and another, there was an overlay of religious belief systems that exacerbated negative attitudes toward both homosexuality and HIV. One participant described this as the perception in some religious communities that HIV was “punishment” for being gay. Another participant noted the impact of colonization and the imposition of dominant Western worldviews that consider sex a taboo subject resulting in barriers to education about HIV in some communities. She noted, “Western philosophy is that you get married and you don’t deal with [sexually transmitted infections]; because of colonization you don’t talk about things … that’s a very taboo subject” (W06).

### The emotional and social impacts of stigma

The system, structural, and social attitudes and behaviours toward people living with HIV had deep emotional impacts. Stigmatizing attitudes toward HIV often resulted in their own feelings of shame and self-stigma. For many participants these feelings were most prominent immediately after diagnosis but could last for years.

Interpersonal and structural oppressions from HIV and intersecting stigmas led to the experience of fear for many participants. Fear was most often related to worries about how a partner, family, or friends would react when they discovered the person’s diagnosis. In many cases people were fearful of violence and rejection. For some, enacted physical and emotional violence led to increased fear of additional and exacerbated violence related to disclosing an HIV diagnosis.

The fear of, or previous experience with, rejection tended to result in deep emotional pain. For many participants, although feelings of shame and internalized stigma dissipated over time, the fear of rejection from sexual partners, family, friends, and acquaintances often persisted. Participants responded to the fear of rejection in various ways such as avoiding intimate relationships in which they felt disclosure might be necessary. Some participants noted that fear of rejection prevented disclosure when there was a desire to talk more openly about HIV to family, friends, and acquaintances.

Social isolation and pervasive feelings of loneliness was a prominent outcome of stigma. Stigma precipitated disruption in previous relationships. Participants talked about losing relationships once their friends or family found out they were HIV-positive. One participant said: “Like nobody won't want to come close to me because what I have, or visit me. I lost a lot of friends because of it … I don’t go around people anymore because they always bully me” (W12). Fear of rejection was often the impetus for long term isolation and loneliness. Several participants talked about their strategies for protecting themselves emotionally by “isolating” themselves, “hiding,” or “building a wall.”

### Striving toward well-being

Despite the many negative impacts of stigma on participants’ lives, they all strove toward well-being in their own ways, and were at various stages of having lives they considered to be good. Participants shared their perspectives about the factors that supported their journey toward well-being in the context of HIV and intersecting forms of stigma. Four themes related to these factors were conceptualized from the data: a) caring for oneself; b) caring for people and pets, c) reconstituting social supports, and d) resisting and disrupting stigma.

#### Caring for oneself

Participants reported that being diagnosed with HIV was a disruption that caused personal distress and was difficult to endure. Those who mentioned this disruption also indicated that they needed time to personally deal with their diagnosis. For one participant, “HIV was the biggest rock thrown at me” (W03). In response to the diagnosis of HIV, some participants stated that they dealt with the diagnosis through behaviours they later saw as self-destructive. For example, one participant noted that there is “devastation finding out that you are HIV positive” and that “it is common you hear people self-medicating, you know like whatever drugs or alcohol and especially the first year, and it [is] usually the hardest year” (W03).

**Fig. 1:**
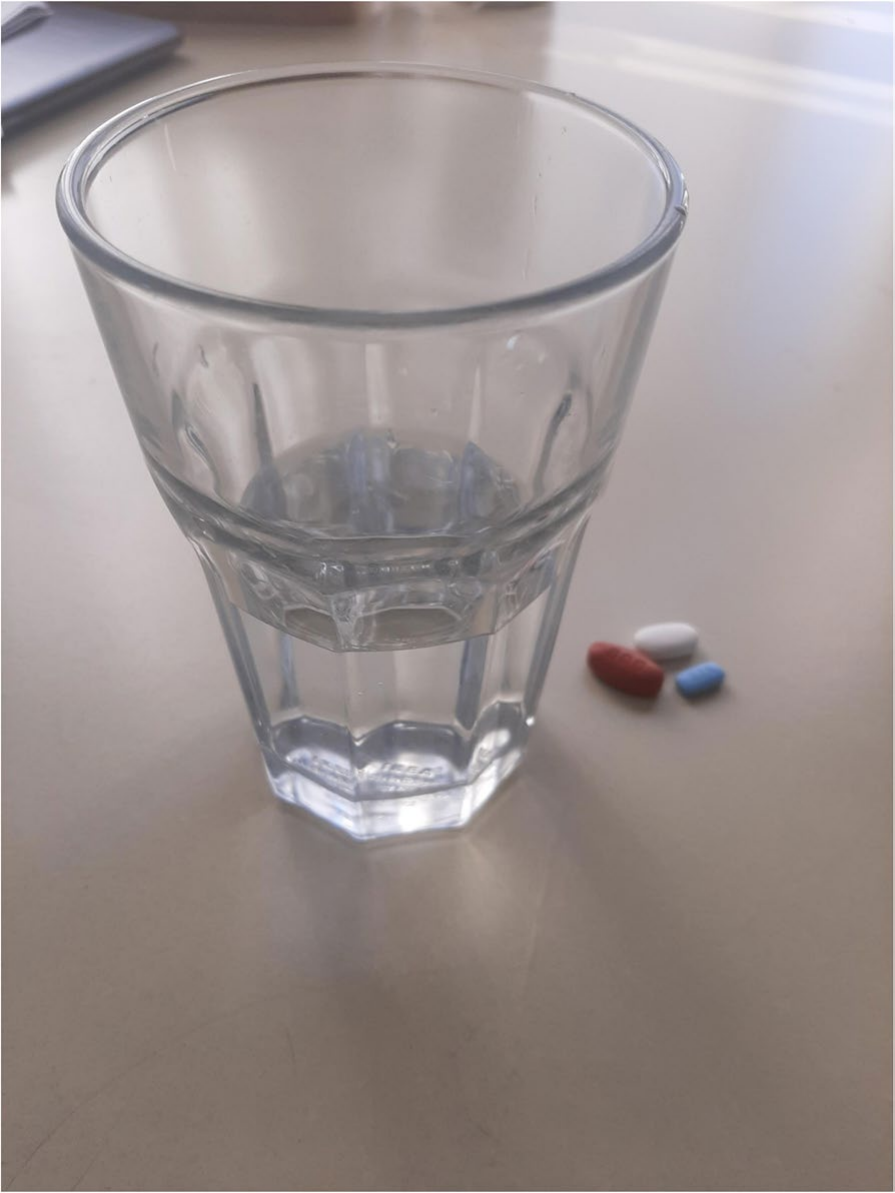
Pills

Many participants spoke about the transition toward looking after themselves physically. This was not always a smooth or direct path but evolved as they gained a greater understanding of how they could live well with HIV. Although some participants talked about taking care of their physical health though good nutrition and exercise, adherence to a strict medication regime was challenging for many. One participant who initially struggled with making HIV medications a part of her life referred to the picture of pills (Fig. 1) stating said,

“I think of this picture, a very powerful picture for people living with HIV, you know, because that’s what you have to remember. You gotta remember that every day, you know…. You gotta have that glass of water. You gotta have those drugs…. And, uh, it’s helping me in my life, my life span” (W01).

Participants talked about the importance of taking care of themselves emotionally. Overcoming the self-stigma that affected their self-esteem was central to improving their emotional well-being. One participant described addressing the anger they felt when they were first diagnosed:

I am totally a different person then I was when I was first diagnosed. I had a lot of anger issues about society, religion, my parents, and my friends, and with this stigma of HIV and through the years, just working with the counsellor has really burst the bubble, the venom that was inside me. I learned to love myself and respect myself (M09).

**Fig. 2:**
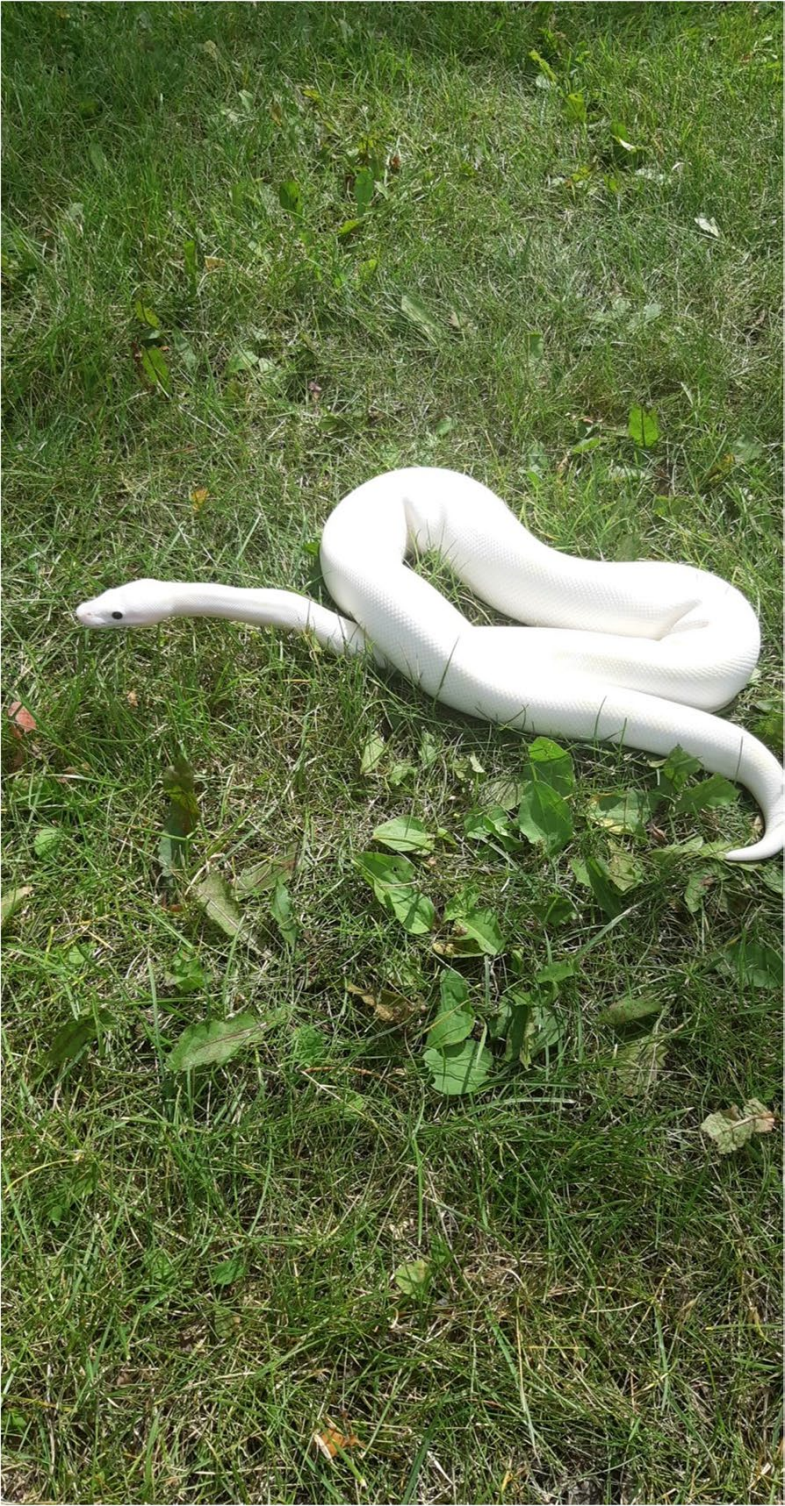
White snake

Another participant echoed a common perspective of the importance of maintaining hope: “I will feel down if I lost hope with myself and about my future living with HIV… I don’t want to lose that hope” (W05). Self-respect and maintaining hope translated into acts of resistance for participants: “I guess the strength is to hold your head up high, even during adversity … and never give in to people’s bigotry and stigma they have against you. Just hold your head up high” (M09). Another participant used the analogy of a snake (Fig. 2) to describe her approach to dealing with stigma.

[The snake] is a powerful creature and he is god’s gift and that’s how I feel about myself and about stigma. Yeah sure it burns and hurts for a little bit and then you get over it and you take everybody’s words and opinions into consideration. If your opinion is ignorant and if it demeans me in anyway, I am not going to take that and carry that negativity with me. I am going to dispose it, just like that snake sheds its skin (W06).

Taking care of oneself emotionally and physically also had spiritual and cultural dimensions in which participants sought “peace” and grounding in particular environments. For some, spending time in nature and the natural environment was important. One participant stated: “I find that nature brings me a lot of peace… I can breathe in the air I can just be in the moment… I find that so makes me feel relaxed” (W03).

Another participant used a photo of a trapline to talk about her hope to live in the wilderness and teach her grandchildren about that way of life. Although she recognized that she needed to be close to an urban centre to access health services, her wish to be connected to nature was a prominent theme in her story.

You won’t hear vehicles going by and you will just hear the leaves dancing around and the water flowing. That is how I would like to live. I know that I have HIV, but I would like to live in the wilderness with my grandchildren (W12).

Some participants found “peace” in spaces that they created for themselves. One participant described a place in his home that he had created and the spiritual value it held for him:

It is a very peaceful place, remembrance of people who have passed on, family and friends that are still alive, and momentums of people and my dogs that passed away.… It is a place that I can be; centre myself with my creator and creatoress. I light the incense and I put the light on, and I mediate a little bit (M09).

Taking care of oneself emotionally and spiritually including cultural aspects of healing, were essential. For one participant participation in a group that took part in sweat lodges, shake tents, drumming and singing was essential to her well-being. She said: “we reach back down to our roots and our ancestry to heal with HIV” (W06).

#### Caring for people and pets

Participants talked about the importance of caring for, and about, people and pets as vital to their own well-being. Several women participants identified the importance of children and mothering in their lives. The knowledge that they could have children who would be HIV-negative was life changing. One participant remembered the hope she felt when she learned she could give birth to a baby who would be HIV-negative: “I am going to love my baby to no end. My baby is going to be born healthy. My baby is going to be born HIV negative, and they are going to grow up, and they are going to have lives of their own” (W07). Another participant talked about her children being the source of her resilience.

**Fig. 3:**
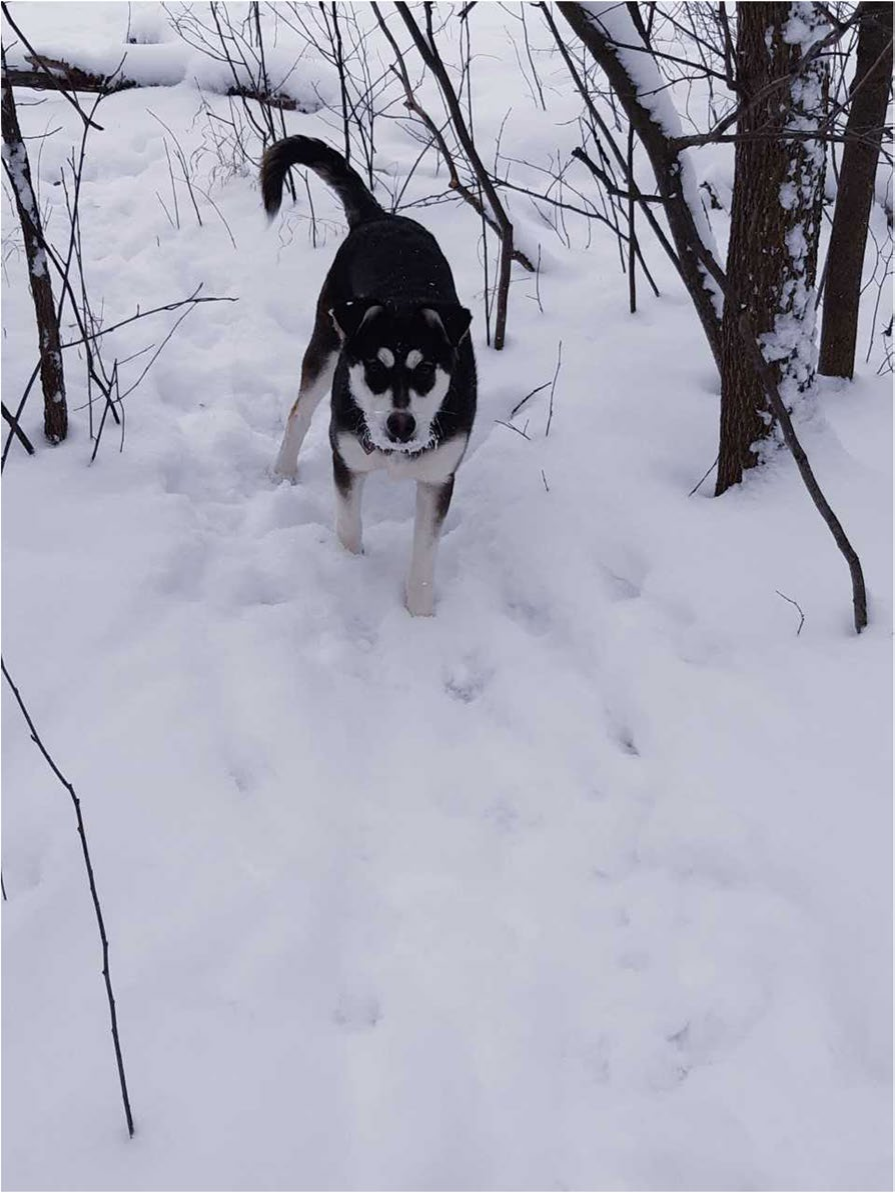
Dog

Some participants noted the mutual caring they experienced from pets, particularly cats and dogs. Pets helped these participants feel less lonely and to enjoy the things that make them feel good, like one participant who talked about going outside for a winter walk—not alone but in company of her dog (Fig. 3).

We go for walks… It makes me feel good because that is something that I really enjoy … and I am not alone, and I don’t have to stifle by having people around me. Sometimes you just need to be alone but not completely alone. Makes me feel good anyways (W03).

Another participant described her cat as being unquestionably supportive “a cat doesn’t care,… doesn’t care if you’re sick… the cat will actually try and take care of you most of the time” (W01).

Some participants also highlighted their responsibility to reduce risks to other people, particularly sexual partners. “We have to be careful; we have to … minimize the risk for other people and that includes me taking my medication. If I didn’t take my medication, I would be a greater risk to society” (M02). One participant used the analogy of driving a car and how the driver is “responsible for the rest of the people in the vehicle” (W01). In addition to reducing risks to people, reciprocity of caring was identified as being very meaningful. Another participant said that people “cannot live individually. We have to support each other; we have to care for each other no matter what…. All human beings [need] to support each other or otherwise it is meaningless” (W05).

#### Reconfiguring social networks

Participants experienced disrupted, lost, and missed opportunities for relationships due to stigma and needed to reconfigure some of their social networks in response. Of particular importance for many participants was connecting with a community of peers who helped relieve the emotional burden of stigma. This ability to find connection could be life-affirming and life changing. One participant noted the importance of:

Meeting other people who are HIV positive, knowing that I was not alone. There are so many people who are dealing with the same thing. I think that was probably what brought me out of it, it just being able to talk with other people (W03).

This peer support assisted her emotionally and gave her the strength to talk openly with her family about being HIV-positive. She viewed disclosure of her HIV-positive status to her family as a major step in moving on with her life.

Finding a network of support was core to many participants’ connection with others and to nurturing a positive sense of self. Participants described peer connections as comforting, as instilling feelings of power, and as providing role models who have prevailed over oppression and discrimination.

#### Resisting and disrupting stigma

Participants resisted stigma in their own lives. Several participants worked diligently to disrupt stigma within their social circles, and a few had become strong advocates for social change. Resisting stigma tended to be a personal journey. As participants learned more about HIV and spent time with people who were accepting and non-judgmental, the oppressive social attitudes and structures that created and sustained stigma and the intersections across racism, sexism, and homophobia became more evident and lessened self-blame.

Although often challenging, some participants spoke about the ways they resisted the stigmas that were directed toward them personally. This included educating people in their social circles, such as family, friends, and acquaintances. Educating other people was sometimes in the context of the disclosure about their own HIV-positive status with the goal of negotiating and preserving relationships. At other times it was motivated by helping people feel more comfortable around them as described by a participant who educated women who were incarcerated about HIV. Another participant spoke about wanting to educate people in her community without disclosing her own status:

Like with my friends, I don’t want to tell them about my stigma, about my HIV but sometimes I try to share [HIV information with] them without telling them [details about me] (W05).

A few participants became strong advocates for social change by speaking publicly about their own HIV experiences. They became peer mentors and spoke in variety of forums, such as workshops and conferences, with the goal of disrupting HIV stigma.

### Resources and supports

The journey toward wellness was not a smooth or direct path. Understanding and supportive partners, families, and friends were essential. Participants recognized the social and structural drivers of stigma and resource availability. They also identified several structural and system supports and resources that they believed were necessary to improve well-being of people living with HIV and disrupt stigmas. These were themed as: a) financial, housing, and food security; b) health, social, and justice services that are trauma-informed, culturally safer and responsive, and HIV evidence-informed; c) anti-oppressive community spaces; d) timely, factual, and universal access to education about HIV; e) access to peer and professional supports related to strategies to address the social and emotional aspects of stigma; and f) training, support, and opportunities to engage in peer mentorship, public education, and anti-stigma advocacy for people living with HIV.

#### Financial, housing, and food security

Several participants spoke about the importance of securing financial resources for housing and food security. Financial insecurity could predate an HIV diagnosis but the increased need for medications and a healthy diet, along with gaps in their ability to work due to illness after an HIV diagnosis, could exacerbate vulnerability. For example, one participant used the photo of a downtown bus shelter as a catalyst to talk about a time when he was hospitalized, couldn’t work, and didn’t have sufficient funds to pay for his rent or the insurance deductible for his medications. He said, “that’s what that image burned in my mind is [I] could have been [sleeping in a bus shelter]; thank [my] lucky stars that [I] didn’t end up totally homeless” (M02). He went on to say that governments need to provide enough funding to prevent homelessness. Several additional participants reinforced the importance of social supports to the well-being of people living with HIV. For example, a woman who identified as an immigrant to Canada noted the role of social supports in meeting the holistic needs of people living with HIV “emotionally, mentally, physically and spiritually.”

#### Health, social, and justice services that are trauma-informed, culturally safer and responsive, and HIV evidence-informed

Several participants spoke about having negative experiences within health, social, and justice systems and how these experiences could increase the trauma of HIV stigma and discrimination. At the time of diagnosis, these experiences included not being allowed to have a friend with them when given the results of their HIV test or not being provided with important information. Experiences also included questions from health and social care providers that the participant felt were stigmatizing. One participant talked about health promotion materials in the waiting room that they felt depicted people with HIV as being from particular racial and ethnic groups. In several cases participants felt that health and social care workers were ill-informed about current evidence related to HIV and that more needed to be done to ensure they were well-educated.

#### Anti-oppressive community spaces

Participants’ stories about their experiences of racism, sexism, homophobia, and HIV-related stigma frequently occurred in schools, churches, health and social care institutions, and other community spaces. The need for strategies to reduce and eliminate oppression in these spaces was a desire of several participants.

#### Timely, factual, and universal access to education about HIV

Like the need for education for workers in health and social service systems, participants talked about the need for more universal access to HIV-related education. This was expressed variously as doing workshops and sessions in locations such as schools to increase the public’s education about HIV and stigma reduction. Participant W03 noted,

We need to address stigma at many levels. There is a stigma in the healthcare system, there is stigma in the general population, there is stigma in employers. Like how do we address that? More education, having that conversation.

Several participants shared the view that conversations about HIV were needed to ensure that the most up-to-date information about HIV was available to people living with HIV as well as the public. Examples of ways that participants felt this universal educational approach would be beneficial included reducing internalized stigma and grief when people are first diagnosed, and increasing employers’ understanding of HIV to facilitate employment opportunities and retention for people living with HIV.

#### Access to peer and professional supports related to strategies to address the social and emotional aspects of stigma

Several participants spoke about the importance of peer supports. One participant described the positive difference that finding peer support in a health clinic made to her. In talking about peers that she met at the clinic she said,

You really just need to be given an HIV mentor when you are first diagnosed… you need someone that lives with it [HIV]. Only they can explain what it’s going to be like for the rest of your life. A doctor can’t tell you what it is like for the rest of your life. But getting to talk to people who live with it and knowing their experiences and how they dealt with it and how they’re doing now. I really could have used that when I first got diagnosed. I really could have used that family in the beginning. I am so grateful that I found them when I needed them (W07).

This quote represented the idea that multiple types of supports were needed that emphasized different perspectives. Important medical needs could be addressed by a doctor while participants also found peer support and professional counselling valuable in addressing social and emotional needs including resisting stigma.

#### Training, support, and opportunities to engage in peer mentorship, public education, and anti-stigma advocacy for people living with HIV

A few participants spoke about their experiences being mentors, educators, and advocates. One participant spoke about the importance of these experiences and the need for more opportunities.

I have come so far since my first diagnosis 10 years ago to where I am. [I am now] going to national conferences and speaking to the media, to being a support system for other Indigenous women or anyone in general with HIV. One of my big concerns is that I would like to see in future… a peer mentorship program where… I can speak to someone… especially newly diagnosed, to normalize it (HIV) and to actually let them know somebody cares (W06).

Although this participant had found opportunities for peer mentorship, education, and advocacy, she also believed that more were needed. These opportunities needed to come with dedicated funding to support activities like a formal peer mentorship program.

## Discussion

Photovoice methodology created an environment in which participants had control over what they chose to talk about when discussing HIV-related stigma by choosing photos as a catalyst for their conversations about stigma. All stories were unique, yet several themes were evident across multiple stories.

All participants talked about the many ways that stigma was deeply embedded with a diagnosis of HIV and had affected them emotionally and socially. Stigma was an added burden compounding the distress of coming to terms with, and adjusting their everyday activities to, living with a chronic and potentially life-threatening health condition. Participants highlighted the impact of shame and internalized stigma associated with HIV on their social and emotional well-being.

The challenge of internalized stigma has been identified in previous research and is prominent for people living with HIV [44]. Internalized stigma has been recognized as originating from the social and structural drivers of stigma pervading society [45] and has been shown to have complex relationships with anticipated stigma [46] and perceived community stigma [26]. For several participants, overcoming internalized stigma was pivotal to living well with HIV. Emlet et al. [47] found in their sample of people with HIV, 50 years and older, that older age was associated with less internalized stigma suggesting that it may be an important aspect of people’s long term stigma management strategies. On the other hand, the negative associations between stigma and well-being can be stronger for older adults, perhaps because of the added effects of ageism [48].

For participants in the current study, enacted and anticipated stigma contributed to fear of rejection and, in some cases, violence. Enacted stigma was experienced by participants in a variety of ways both interpersonally and structurally. Examples included women participants who experienced new or increased intimate partner violence related to their HIV status, and gay men who experienced community violence. Participants also experienced rejection from family and friends or anticipated that they would, a finding consistent with participants in Reinius et al.’s [49] study. These experiences of enacted and anticipated stigma prompted some participants to leave relationships or avoid situations in which additional rejection or violence related to HIV stigma was anticipated. These avoidance experiences could be a highly adaptive response to cope with extremely difficult situations [7]. However, this response can also result in the loss of important opportunities for life affirming social support networks.

For participants in the current study internalized, enacted, and anticipated HIV stigma intersected with additional stigmas in complex ways to contribute to disrupted, lost, and missed opportunities for relationships and social support networks. Loneliness was a common outcome and previous research has suggested a complex relationship among HIV stigma, loneliness, and depression [50], and reduced social support [7]. Rejection, social isolation, and the loss of social supports can have negative effects on people’s health [26] and has been shown to be a barrier to HIV treatment and care [51] including adherence to antiretroviral treatment [25].

Intersectionality of HIV-related stigma with additional stigmas was a prominent theme in the current study. Building on the work of Crenshaw [52], Logie and colleagues described intersectional stigma as the “interdependent and mutually constitutive relationship between social identities and structural inequities” ([53],p.9). Participants in the current study spoke about HIV-related stigma at the intersections of additional forms of oppression. Consistent with previous research, intersectionality was experienced related to race [22, 53–55], sex and gender [53, 54], and sexuality [55]. These additional oppressions were often experienced prior to an HIV diagnosis and many participants perceived that being diagnosed with HIV compounded their experiences of oppression. Earnshaw and colleagues [56] found that internalized stigmas related to race and sexual orientation before HIV diagnosis were associated with greater HIV-related internalized stigma after diagnosis. This suggests both the independent and compounding effects of intersecting stigmas. The implication is that, to improve the well-being of people living with HIV, the social systems and structures that perpetuate stigmas of all forms need to be addressed at their common root causes, including coloniality. Intersectionality is an important consideration in studies on HIV stigma and we support calls for additional attention to this research [57, 58]. Ongoing anti-racism interventions and the development of culturally safer spaces must consider intersecting identities.

Participants talked about the ways they adapted to a life with HIV in the context of pervasive social stigma. Caring for themselves was a way of addressing the physical, mental health, and spiritual challenges of living with a stigmatized chronic health condition. Good nutrition, exercise, and medication adherence were important to maintaining physical health. Looking after their own emotional and spiritual health were important as well. Maintaining hope and working to increase self-respect in the context of internalized stigma were important parts of this journey. Finding places that could help them find peace were often, but not always, in nature. For some, as previously identified by Indigenous scholars (e.g., [59]), culturally-based healing practices were instrumental to promoting well-being. Attention to culture and spirituality has long been neglected in Western Eurocentric health care and our findings suggest that this needs to change. Respectful collaboration with communities is essential. For example, collaboration with communities in the design and implementation of HIV-related health care is important in providing relevant health education and care targeted to African, Caribbean, and Black people [60]. Community involvement and leadership can increase access of Indigenous people to culturally responsive health programs such as land-based healing [61].

Having a person or an animal to care for, and about, was an important part of some participants’ lives. Not surprisingly, caring for children emerged has having special significance. In addition, caring for pets and having the companionship of pets was perceived as being important to emotional well-being. Previous research has also highlighted the positive effects of pet ownership for people with HIV [62–64]. Pets can provide comfort, a sense of purpose, and a buffer against stress and loneliness. Health and social care providers should be aware of the potential benefit for some people living with HIV.

Reconstituting social networks was an important process for many participants in their journey toward greater wellness. There was particular emphasis on connecting with people who were also living with HIV. Participants in the present study discussed the importance of peer support, often in the context of a support group. They identified the value of peers in providing information that helped them to live with HIV and supporting their positive life choices such as negotiating disclosure decisions with friends and family. A recent systematic review of randomized controlled trials found evidence for the positive effects of peer support in clinical care cascade outcomes, particularly retention in care, medication adherence, and viral suppression [65]. There was inconclusive, yet promising, evidence for positive effects on mental health outcomes. The current qualitative study, points to the importance of peer support for emotional, and in some cases, spiritual, well-being.

Resisting and disrupting stigma in their own lives and in the wider population were important processes for participants in the current study. They identified structural and social supports that can promote stigma reduction and increase opportunities to flourish. We agree with Nyblade et al. [5] that, to end the AIDS epidemic, stigma reduction needs to be the highest priority. Participants in the present study provided guideposts for action. Some of these actions will be relatively easy to implement while others are considerably more difficult and will require concerted system and policy contributions.

Access to peer and professional support to address the personal and emotional effects of HIV and associated stigma must be a priority. Programs of research focused on treatment are important; yet reducing stigma and the emotional impacts of stigma continues to be neglected as a source of well-being and a pathway to adherence to medical interventions. Newly diagnosed individuals need access to supports that buffer the effects of stigma. Participants in the current study emphasized the importance of peers with whom they strongly identified (e.g., Indigenous women living with HIV), and/or for whom there was a sustained connection such as in an ongoing support group or relationship. Associated with implementation of peer supports is the development of training, support, and opportunities for people living with HIV to become peer mentors, public educators, and anti-stigma advocates. Timely, factual, and universally accessible public education about HIV needs to be a priority. These interventions can be implemented relatively easily once they are considered essential to wholistic care.

Participants noted the oppressions they experienced in various organizations and in public and private spaces. Promoting anti-oppressive community organizations and spaces is essential. This finding draws attention to the anti-oppression role of religious, cultural, recreational, educational, employment, and additional public and private organizations and environments where people meet, gather, and contribute to the social fabric of society. Importantly, health, social, and justice services need to become universally trauma-informed [66], culturally safer [67, 68], and HIV evidence-informed to resist and disrupt all forms and intersections of stigma, discrimination, and oppression.

Addressing the root structural socio-political drivers that limit people’s ability to flourish while living with HIV will have a great impact. Directly related to HIV stigma, participants highlighted the essential need for people to have financial, housing, and food security to resist stigma and live a good life. These largely neglected structural and system interventions require resources and commitments that recognize their essential contribution to people flourishing while living with HIV.

### Limitations and strengths

As a case study, this research may be limited in its application to other contexts and jurisdictions. The small sample size meant that we did not have perspectives of people with a full range of gender, racial, and ethnic identities. Recruitment efforts were hampered by COVID-19 pandemic lockdowns. Although sample sizes in photovoice studies vary considerably, our sample of 11 participants was slightly above the 6 to 10 range recommended by Wang & Burris [32].

Photovoice methodology was limited by the need to collect data individually due to COVID-19 pandemic lockdowns reducing opportunities for collective discovery and action planning among the participants as a group. However, photovoice methodology gave participants the tools to choose what they, as individuals, felt was important in telling their stories of stigma and how they built resistance to stigma over time. Despite variations of experiences and responses there were patterns that suggest important actions that can reduce stigma and its impacts on people living with HIV.

Participants highlighted intersections of HIV stigma with stigmas related to race, sex and gender, and sexual orientation. Other research has identified intersections with additional social identities and inequities including substance use [69], poverty [70], shelter insecurity [22], illness [55], disability [71], and roles and expectations [55]. Although these additional stigmas may have been experienced by, and affected, participants, the nature of the photovoice methodology meant that participants identified the stigmas and intersections that they themselves prioritized in the interviews. These priorities may have been influenced by the location and timeframe of the interviews, the characteristics of the participants, and the significance of the particular stigma to the participant. Intravenous drug use is a risk factor for HIV transmission in the jurisdiction in which this study took place, yet none of the participants spoke specifically about experiences of intravenous drug use or its associated stigmatization at the intersection of HIV. However, the clear message from participants in this study is that it is important for practitioners, policy makers, and researchers to consider the intersectionality of stigmas, whatever their source, in all of their work.

## Conclusion

Participants’ experiences of their journeys toward living well within the context of HIV-related stigma were unique and an ongoing process. However, the results of this research suggest important patterns. HIV-related stigma is pervasive and intersects with additional stigmas to compound the ways that stigma is experienced in enacted, anticipated, and internalized dimensions. These experiences profoundly affect health and social outcomes for people living with HIV and society. The transitions from internalizing to resisting stigma can be expedited by people’s personal assets and supported by health and social service system resources that extend beyond medical care to address basic needs for food, shelter, income, and positive social and community connections. To achieve goals of people living well with HIV and ending the AIDS epidemic, increased health care, social, and policy attention needs to be paid to reducing the foundational system and structural drivers of HIV and intersecting stigmas.

## Data Availability

The datasets generated and analyzed during the current study are not publicly available due the personal nature of the narrative stories obtained through the interviews which makes ensuring anonymity and confidentiality of participants extremely difficult. Some collated data may be available from the corresponding author on reasonable request.
